# Differentiating scrub typhus meningitis from brucellar meningitis in children: A single-center retrospective study

**DOI:** 10.1371/journal.pntd.0014107

**Published:** 2026-03-13

**Authors:** Yonghan Luo, Yan Guo, Ying Zhu, Haifeng Jin, Penghao Cui, Ruonan Li, Qingping Tang, Yin Li, Yanchun Wang

**Affiliations:** 1 Second Department of Infectious Disease, Kunming Children’s Hospital (Children’s Hospital Affiliated to Kunming Medical University), Kunming, Yunnan, China; 2 Faculty of Life Science and Technology, Kunming University of Science and Technology, Kunming, Yunnan, China; 3 Department of Reproductive Gynecology, NHC Key Laboratory of Healthy Birth and Birth Defect Prevention in Western China First People’s Hospital of Yunnan Province, Kunming, Yunnan, China; 4 Department of Reproductive Gynecology, The Affiliated Hospital of Kunming University of Science and Technology, Kunming, Yunnan, China; University of Calgary, CANADA

## Abstract

**Objective:**

To investigate the clinical distinctions between scrub typhus meningitis and brucellar meningitis in children, and to identify potential biomarkers with early differential diagnostic value to support clinical decision-making.

**Methods:**

A retrospective analysis was conducted on 13 pediatric patients diagnosed with brucellar meningitis admitted to Kunming Children’s Hospital over the past decade. Thirteen cases of scrub typhus meningitis were selected as controls using an age- and sex-matching strategy. The clinical manifestations, laboratory findings, and cerebrospinal fluid (CSF) characteristics were compared between the two groups. Receiver operating characteristic (ROC) curve was employed to assess the diagnostic performance and clinical utility of key biomarkers.

**Results:**

The pre-admission fever and duration of fever were significantly shorter in the scrub typhus meningitis group. Laboratory evaluation revealed that serum ferritin, procalcitonin (PCT), C-reactive protein (CRP), and creatinine (Cr) levels were markedly higher in the scrub typhus group compared with the brucellosis group. No statistically significant differences were observed in CSF biochemical parameters. ROC analysis demonstrated that ferritin (AUC = 0.870) and PCT (AUC = 0.846) exhibited the greatest diagnostic accuracy, followed by CRP (AUC = 0.814) and Cr (AUC = 0.799). All patients achieved complete clinical recovery following standardized treatment, with no recurrences or fatalities.

**Conclusions:**

Although scrub typhus meningitis and brucellar meningitis share considerable clinical overlap in children, serum ferritin and PCT levels may represent potential diagnostic signals for early differential diagnosis, warranting validation in larger prospective cohorts. High Ferritin levels or PCT levels may provide preliminary clues toward scrub typhus meningitis. Early recognition and targeted antimicrobial therapy are associated with favorable prognostic outcomes.

## Author summary

In this study, we focus on distinguishing between childhood scrub typhus (ST) and brucella meningitis. Given the numerous clinical similarities between these two diseases, accurate early diagnosis is crucial for effective clinical treatment. Through a retrospective analysis of 13 cases of brucella meningitis over a ten-year period at Kunming Children’s Hospital, and employing propensity score matching, we selected 13 cases of ST meningitis as a control group. Our findings revealed that the duration of fever in the ST meningitis group was shorter, while laboratory tests showed significantly higher levels of serum ferritin, procalcitonin (PCT), C-reactive protein (CRP), and creatinine (Cr) in the ST group compared to the brucella group. Serum ferritin and PCT levels demonstrated potential high diagnostic accuracy in ROC curve analysis for distinguishing between the two forms of meningitis. Our research suggests that serum ferritin and PCT levels may serve as important early biomarkers for differentiating these two types of meningitis, aiding clinicians in making more accurate diagnoses and supporting the development of treatment strategies.

## Introduction

Brucellosis is a zoonotic infectious disease caused by Brucella species, transmitted primarily through direct contact with infected animals or their secretions, or by consuming unpasteurized dairy products [1]. The disease presents with a wide spectrum of clinical manifestations, including fever, night sweats, fatigue, and joint or bone pain [2]. In China, the epidemiology of brucellosis demonstrates marked regional variation, with the highest incidence observed in northern pastoral provinces. However, due to population mobility and the expansion of animal husbandry, sporadic cases have increasingly been reported in non-endemic areas [3]. Although Yunnan Province has not traditionally been a high-incidence region for brucellosis, recent years have seen a rising trend in reported cases, including among children [4].

Scrub typhus (ST), an acute febrile illness caused by Orientia tsutsugamushi, a member of the Rickettsiaceae family, is transmitted through the bites of infected larval mites. It is a natural focal disease predominantly distributed within the so-called “ST triangle,” encompassing the vast region between northern Japan, northern Australia, and Pakistan [5, 6]. Southern China is one of the major endemic zones, and Yunnan Province, located in the Southeast Asian monsoon climate region, is recognized as a key endemic area [7]. The number of pediatric ST cases in Yunnan increases significantly during the summer and autumn seasons.

Both brucellosis and ST can present in children as infections with insidious onset, prolonged fever, and nonspecific systemic symptoms [4, 7]. Although some clinical features can assist differentiation, such as the typical eschar in ST and osteoarticular pain or a history of animal exposure in brucellosis, a substantial proportion of pediatric cases lack these characteristic signs. Studies [8] have shown that approximately 20% of pediatric ST cases may occur without eschar, while one study [9] also noted that about 50% cases did not exhibit joint pain. When epidemiological clues such as animal exposure or outdoor activity are not typical, it is often difficult to distinguish between the two diseases based solely on clinical manifestations. Such diagnostic uncertainty may lead to misdirected antimicrobial therapy (e.g., delayed initiation of doxycycline for ST or inappropriate avoidance of combination therapy for brucellosis), which in turn can prolong illness, increase the risk of complications, and negatively affect clinical outcomes. Therefore, improving early differentiation between the two diseases is clinically important.

Meningitis is a serious complication that can occur in both infections. brucellar meningitis usually indicates a complicated disease course, requires prolonged treatment often exceeding three months, and carries a risk of neurological sequelae or even death [10, 11]. ST meningitis can also lead to severe central nervous system damage [12, 13]. Although cerebrospinal fluid (CSF) examination is commonly performed in both conditions, the CSF findings are generally nonspecific. In addition to elevated cell counts, biochemical changes are usually mild and differ from those seen in typical bacterial or tuberculous meningitis [14]. Pathogen-specific diagnostic methods, such as CSF culture or molecular detection, remain the gold standard for confirmation but are time-consuming and have limited positivity rates, making them insufficient for early differential diagnosis and clinical decision-making [15].

To date, comparative studies focusing on pediatric brucellar meningitis and ST meningitis remain scarce. Therefore, this study retrospectively analyzed brucellar meningitis cases admitted to Kunming Children’s Hospital over the past decade. Using the propensity score matching method, corresponding cases of ST meningitis were selected for comparison. We hypothesize that specific inflammatory and biochemical biomarkers, particularly ferritin and PCT, differ significantly between ST meningitis and brucellar meningitis in children, and that these biomarkers may aid early clinical differentiation of the two conditions. By systematically analyzing the clinical characteristics, laboratory parameters, and CSF profiles of the two groups, this study aimed to identify readily obtainable early diagnostic indicators with significant discriminatory value, thereby providing clinicians with evidence-based support for the differential diagnosis of ST meningitis and brucellar meningitis in children.

## Materials and methods

### Ethics statement

Although informed consent was waived due to the retrospective nature of the study, this study was approved by the Ethics Review Committee of Kunming Children’s Hospital(2024-06-047-k05). Furthermore, we ensured that all patient data were anonymized to maintain confidentiality and privacy, in accordance with ethical standards. This study was carried out in accordance with the ethical standards of the Declaration of Helsinki.

### Consent for publication

Not applicable.

### Study population

Clinical data were collected from 13 hospitalized patients diagnosed with brucellar meningitis, and an additional 13 cases of ST meningitis were selected using an age- and sex-matching strategy. This study was reviewed and approved by the Ethics Committee of Kunming Children’s Hospital.

### Inclusion and exclusion criteria

#### Inclusion criteria for ST.

Diagnosis was confirmed based on the Pediatric Infectious Diseases diagnostic criteria for ST (5th edition) [16]. The clinical diagnosis of ST was established for patients who met the following criteria:

a) A history of outdoor exposure in endemic regions within three weeks prior to symptom onset, presenting with an acute high fever and the characteristic eschar or ulcer, rash, lymphadenopathy, and other typical clinical signs.
b) At least one positive laboratory test result, including: a positive specific IgM antibody test, a fourfold increase in serum antibody titers as confirmed by the Weil-Felix test, or detection of the pathogen through polymerase chain reaction (PCR).

Importantly, in this study, all ST meningitis cases were confirmed by PCR testing, and PCR positivity was required for inclusion. No cases were diagnosed based solely on IgM.

#### Exclusion criteria for ST.

a) Presence of underlying medical conditions such as congenital heart disease, hematological malignancies, or immunodeficiency.
b) Insufficient or incomplete clinical data.
c) CSF analysis showed a normal cell count.

#### Inclusion criteria for brucellosis.

Diagnosis of brucellosis was made based on clinical features and confirmatory diagnostic criteria as outlined in the “Diagnosis and Treatment Scheme for Brucellosis (2023 Edition)” [13]. The inclusion criteria for brucellosis were as follows:

1. Etiological Diagnosis:

Isolation of *brucella* from any pathological specimen, including blood, bone marrow, CSF, pus, or other body fluids/excretions.

1. Initial Screening Tests:
  a) A positive result from the Rose Bengal Test (RBT).
  b) A positive result from the Colloidal Gold Immunochromatographic Assay (GICA).
  c) A positive result from the Enzyme-Linked Immunosorbent Assay (ELISA).
2. Confirmatory Tests:
  a) A positive Tube Agglutination Test (SAT) with a titer of 1:100 or higher, or in cases with persistent symptoms for more than one year, a titer of 1:50 or higher.
  b) A positive Complement Fixation Test (CFT) with a titer of 1:10 or higher.
  c) A positive Coombs Test with a titer of 1:160 or higher.

Clinical Diagnosis: Suspected cases that showed any positive result in initial screening tests.

Confirmed Diagnosis: Cases that, following clinical evaluation, were confirmed by any positive result in etiological diagnosis or serological confirmatory tests.

Importantly, in the present study, only patients who met the criteria for Confirmed Diagnosis were included. Screening-positive suspected cases were not enrolled.

#### Exclusion criteria for brucellosis.

a) Co-infection with other acute infectious pathogens.
b) Insufficient or incomplete clinical data.

Presence of underlying medical conditions such as congenital heart disease, hematological malignancies, or immunodeficiency.

c) CSF analysis showed a normal cell count.

A summary of the diagnostic criteria applied for ST and brucellosis in this study is provided in **S1 Table**. For patients with a prolonged fever lasting over a week or presenting neurological symptoms, such as vomiting, headache, or lethargy, which are indicative of a potential central nervous system infection, a lumbar puncture was performed. Because lumbar puncture was performed on the day of admission due to prolonged fever and suspected central nervous system involvement, the variable “pre-admission fever duration” reflects the interval from symptom onset to lumbar puncture for all patients. Meningitis was considered confirmed when the CSF white-cell count exceeded 10 × 10⁶/L, which is a commonly used clinical threshold for defining CSF pleocytosis.

### Data collection

The dataset included general patient information, such as age, gender, and residence type (rural or urban). Clinical symptoms and signs were systematically recorded, including fever duration, presence of cough, headache, vomiting, abdominal pain, rash, lymphadenopathy, hepatomegaly, and splenomegaly. Both pre-admission fever duration and total fever duration were documented. Laboratory parameters consisted of white blood cells (WBC), platelet count (PLT), hemoglobin (Hb), alanine aminotransferase (ALT), aspartate aminotransferase (AST), albumin (ALB), and kidney function markers. Inflammatory markers including C-reactive protein (CRP), procalcitonin (PCT), and ferritin were measured. Lactate dehydrogenase (LDH) and creatine kinase-MB (CK-MB) values were also obtained, along with coagulation parameters such as activated partial thromboplastin time (APTT), prothrombin time (PT), and fibrinogen (FIB). CSF analysis was performed for all enrolled patients, with key variables encompassing CSF cell count, CSF glucose, CSF protein, and CSF chloride levels. Finally, treatment outcomes for all patients were documented, with particular attention to whether clinical improvement was achieved by the time of discharge.

All peripheral blood samples used for biomarker analysis were obtained within 24 hours of hospital admission and prior to the initiation of in-hospital antimicrobial or corticosteroid therapy. Blood sampling was performed in close temporal proximity to diagnostic lumbar puncture in routine clinical practice.

### Statistical methods

Data analysis was performed using R software (version 4.4.1). The following R packages were utilized for specific analyses: the *MatchIt* package for propensity score matching, the *pROC* package for receiver operating characteristic (ROC) analysis. Continuous variables with a normal distribution were compared using the t-test and are presented as mean ± standard deviation (mean ± SD). Non-normally distributed data were analyzed using the Mann-Whitney U test, and are expressed as median and interquartile range [M (P25, P75)]. Categorical variables were assessed using the chi-square test or Fisher’s exact test, with results reported as counts (n) and percentages (%).

Univariate analysis was conducted to identify factors differentiating ST meningitis from brucellar meningitis. The group differences of these factors were visualized using bar charts. ROC curve analysis was employed to evaluate the discriminatory ability of these factors in differentiating ST meningitis from brucellar meningitis. The ROC curve illustrates the trade-off between sensitivity and specificity across different diagnostic thresholds. In addition, stratified ROC analyses were conducted according to fever-duration strata (≤14 days vs > 14 days) to assess the stability of diagnostic performance across different stages of illness. Statistical significance was set at p < 0.05.

## Results

### Basic Information

Following the application of inclusion and exclusion criteria, and propensity score matching with case controls, this study analyzed 13 pediatric cases diagnosed with ST meningitis and 13 cases diagnosed with brucellar meningitis **(seeFig 1).** Seasonal distribution showed that ST meningitis cases mainly occurred in summer (10 cases) and autumn (3 cases), whereas brucellar meningitis cases occurred in spring (3 cases), summer (7 cases), autumn (2 cases), and winter (1 case).

**Fig 1:**
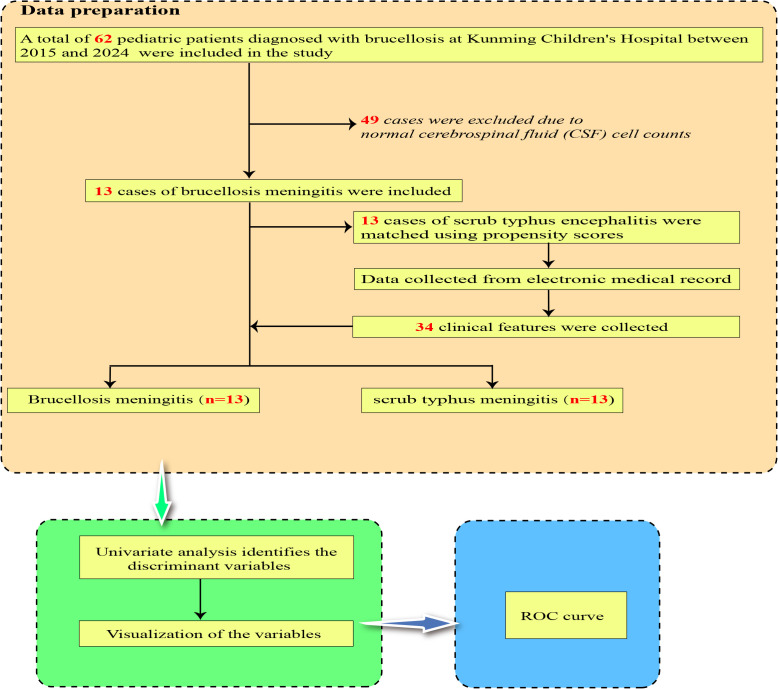
Data preparation and analytical workflow of the study. receiver operating characteristic (ROC).

The average age of the patients was 5.18 ± 3.63 years. The mean age of the ST meningitis group (6.22 ± 4.3 years) was higher than that of the brucellar meningitis group (4.14 ± 2.59 years), though the difference between groups was not statistically significant (p = 0.151). The gender distribution was similar between the two groups, with males comprising 62% and females 38% of the total sample. No significant gender differences were found between the groups (p = 0.687). The majority of patients were from rural areas (96%), and there was no statistically significant difference in the rural vs. urban distribution between the groups (p = 1) **(see Table 1)**.

**Table 1:** Comparison of clinical characteristics between scrub typhus meningitis and brucella meningitis.

Variables	Total (n = 26)	Brucellar meningitis (n = 13)	ST Meningitis (n = 13)	p-value
**Basic Information**				
Age, years, Mean ± SD	5.18 ± 3.63	4.14 ± 2.59	6.22 ± 4.3	0.151
Gender, n (%)				0.687
Female	10 (38)	6 (46)	4 (31)	
Male	16 (62)	7 (54)	9 (69)	
Rural Residence, n (%)				1
NO	1 (4)	1 (8)	0 (0)	
YES	25 (96)	12 (92)	13 (100)	
**Symptoms and Signs**				
Pre-admission Fever, days, Median (Q1, Q3)	10.5 (8, 17.25)	18 (10, 30)	10 (8, 11)	0.064
Fever Duration, days, Median (Q1, Q3)	15 (10.25, 20.75)	21 (12, 34)	13 (9, 15)	0.019
Fever, n (%)				1
YES	26 (100)	13 (100)	13 (100)	
Cough, n (%)				0.097
NO	17 (65)	11 (85)	6 (46)	
YES	9 (35)	2 (15)	7 (54)	
Headache, n (%)				0.673
NO	18 (69)	10 (77)	8 (62)	
YES	8 (31)	3 (23)	5 (38)	
Vomiting, n (%)				0.593
NO	22 (85)	12 (92)	10 (77)	
YES	4 (15)	1 (8)	3 (23)	
Abdominal Pain, n (%)				0.322
NO	21 (81)	12 (92)	9 (69)	
YES	5 (19)	1 (8)	4 (31)	
Rash, n (%)				1
NO	19 (73)	9 (69)	10 (77)	
YES	7 (27)	4 (31)	3 (23)	
Lymphadenopathy, n (%)				1
NO	20 (77)	10 (77)	10 (77)	
YES	6 (23)	3 (23)	3 (23)	
Hepatomegaly, n (%)				0.673
NO	18 (69)	10 (77)	8 (62)	
YES	8 (31)	3 (23)	5 (38)	
Splenomegaly, n (%)				1
NO	17 (65)	9 (69)	8 (62)	
YES	9 (35)	4 (31)	5 (38)	
**Laboratory Tests**				
WBC, × 10⁹/L, Median (Q1, Q3)	9.18 (7.2, 11.73)	8.22 (6.05, 8.74)	10.47 (9.62, 17.9)	0.014
PLT, × 10⁹/L, Mean ± SD	218 ± 110.35	255.62 ± 87.83	180.38 ± 120.79	0.083
Hb, g/L, Mean ± SD	117.73 ± 17.64	117.23 ± 14.24	118.23 ± 21.1	0.889
ALT, U/L, Median (Q1, Q3)	35.45 (24.5, 50.75)	28 (22, 38)	44 (26, 96)	0.166
AST, U/L, Median (Q1, Q3)	47 (35.5, 67)	42 (40, 63)	50 (34, 69)	0.626
ALB, g/L, Median (Q1, Q3)	36.55 (30.32, 41.62)	39 (35.5, 42.5)	30 (27, 39.5)	0.072
CK-MB, U/L, Median (Q1, Q3)	19 (14.88, 22)	19 (16, 22)	16 (14, 21)	0.44
LDH, U/L, Median (Q1, Q3)	363.5 (270.75, 502.75)	312.7 (269, 385.2)	445 (319, 626)	0.169
Cr, μmol/L, Mean ± SD	27.98 ± 10.65	22.87 ± 6.86	33.09 ± 11.52	0.013
FIB, g/L, Mean ± SD	2.93 ± 0.84	3 ± 0.44	2.87 ± 1.13	0.692
APTT, s, Mean ± SD	38.06 ± 9.9	36.92 ± 7.36	39.2 ± 12.13	0.569
PT, s, Median (Q1, Q3)	13 (12.55, 14)	13 (12.5, 13.5)	13 (12.7, 14)	0.918
CRP, mg/L, Median (Q1, Q3)	9.05 (1.96, 30.93)	2.37 (1.18, 6.4)	30.11 (11.65, 47)	0.007
PCT, ng/mL, Median (Q1, Q3)	0.5 (0.26, 0.82)	0.29 (0.25, 0.5)	0.88 (0.5, 2.38)	0.003
Ferritin, μg/L, Median (Q1, Q3)	193.85 (134.27, 435.5)	143 (116, 165)	468 (242, 579)	0.001
**Cerebrospinal Fluid (CSF)**				
CSF Cell Count, × 10⁶/L, Median (Q1, Q3)	72 (17.25, 159.75)	85 (14, 106)	65 (22, 169)	0.521
CSF Glucose, mmol/L, Mean ± SD	2.96 ± 0.75	2.87 ± 0.72	3.04 ± 0.8	0.585
CSF Protein, g/L, Median (Q1, Q3)	0.17 (0.11, 0.32)	0.12 (0.1, 0.22)	0.2 (0.16, 0.42)	0.086
CSF Chloride, mmol/L, Mean ± SD	123.09 ± 4.68	123.24 ± 4.25	122.95 ± 5.26	0.877
**Outcome**				1
Improved, n (%)	26 (100)	13 (100)	13 (100)	

### Clinical symptoms and signs

All patients exhibited fever (100%), with the duration of fever being significantly shorter in the ST meningitis group compared to the brucellar meningitis group (13 days vs. 21 days, p = 0.019). The ST meningitis group showed a higher proportion of cough (54% vs. 15%), but the difference was not statistically significant (p = 0.097). Headache, vomiting, abdominal pain, rash, lymphadenopathy, and hepatosplenomegaly were observed in both groups, though the incidence of these symptoms did not differ significantly (p > 0.05).

### Laboratory tests

Laboratory results revealed that the WBC was significantly higher in the ST meningitis group (10.47 × 10⁹/L vs. 8.22 × 10⁹/L, p = 0.014; reference range: 4–10 × 10⁹/L). Additionally, serum creatinine (Cr) levels were significantly elevated in the ST meningitis group (33.09 μmol/L vs. 22.87 μmol/L, p = 0.013; reference range: approximately 20–60 μmol/L). CRP was notably higher in the ST meningitis group (30.11 mg/L vs. 2.37 mg/L, p = 0.007; reference range: < 5 mg/L), and PCT levels were also significantly higher (0.88 ng/mL vs. 0.29 ng/mL, p = 0.003; reference range: < 0.1 ng/mL). Ferritin levels were substantially higher in the ST meningitis group (468 μg/L vs. 143 μg/L, p = 0.001; reference range: 10–150 μg/L). No significant differences were observed between the groups for other laboratory markers, such as Hb, PLT, ALT, or AST (p > 0.05).

### Cerebrospinal fluid analysis

CSF analysis revealed no significant differences between the two groups in terms of CSF cell count glucose CSF glucose, protein CSF Protein, or chloride levels CSF chloride (p > 0.05).

### Outcomes

Regarding clinical outcomes, all patients showed clinical improvement with no relapses (100% recovery rate).

### Diagnostic value of variables in differentiating ST meningitis from brucella meningitis

**Fig 2:**
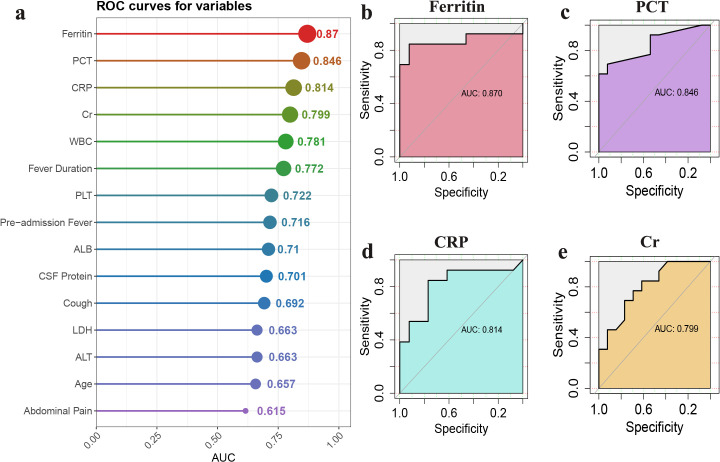
Receiver Operating Characteristic (ROC) curve analysis of clinical variables. **(a)** Comparison of the area under the ROC curve (AUC) for each clinical variable in distinguishing Brucellosis and Scrub Typhus meningitis. **(b–e)** ROC curves for each individual marker: **(b)** Ferritin, **(c)** Procalcitonin, **(d)** C-Reactive Protein, **(e)** Creatinine. The x-axis represents 1–specificity, and the y-axis represents sensitivity, with each curve reflecting the diagnostic performance of the respective marker in differentiating Brucellosis and Scrub Typhus meningitis.

We first employed receiver operating characteristic ROC curve analysis to evaluate the diagnostic performance of various clinical and laboratory parameters in distinguishing ST meningitis from Brucella meningitis **(Fig 2a–2e).**

The results revealed that ferritin, PCT, CRP, and Cr exhibited high predictive accuracy. Among the evaluated biomarkers, ferritin exhibited the highest diagnostic performance, with an area under the ROC curve (AUC) of 0.870 (95% CI: 0.703–0.943). This was followed by PCT, with an AUC of 0.846 (95% CI: 0.693–0.958), CRP with an AUC of 0.814 (95% CI: 0.635–0.941), and serum creatinine, with an AUC of 0.799 (95% CI: 0.628–0.929). In addition, WBC, duration of fever, and platelet count demonstrated moderate discriminatory ability. Other variables, including CSF protein, ALB, cough, LDH, ALT, age, and abdominal pain, exhibited lower diagnostic performance **(Fig 2a). Fig 2b–2e** further visualized the ROC curves of the four most diagnostically valuable biomarkers.

Given the limited sample size, Decision Curve Analysis was not performed. Instead, conventional diagnostic performance metrics were calculated for key biomarkers using cut-off values derived from the Youden index (exploratory). Using a cut-off value of 219.6 ng/mL, ferritin demonstrated a sensitivity of 84.6% and specificity of 92.3%, with a positive likelihood ratio (LR+) of 11.0 and a negative likelihood ratio (LR−) of 0.17. Using a cut-off of 0.505 ng/mL, PCT showed a sensitivity of 69.2% and specificity of 92.3% (LR + 9.0, LR − 0.33). CRP, at a cut-off of 6.425 mg/L, yielded a sensitivity of 84.6% and specificity of 76.9%, whereas serum creatinine, at a cut-off of 23.3 µmol/L, showed a sensitivity of 84.6% and specificity of 61.5%.

To further assess whether fever duration influenced the discriminatory performance of these biomarkers, stratified ROC analyses were conducted according to fever-duration strata (≤14 days vs > 14 days). Within both strata, ferritin, PCT, CRP, and Cr consistently retained discriminatory ability for ST meningitis. In the ≤ 14-day stratum, ferritin, PCT, CRP, and Cr yielded AUCs of 0.813, 0.891, 0.891, and 0.750, respectively. In the > 14-day stratum, the corresponding AUCs were 0.998, 0.867, 0.911, and 0.822. These findings indicate that the direction and magnitude of diagnostic performance remained generally consistent across fever-duration strata, suggesting that the observed associations were not solely attributable to differences in timing of presentation. However, given the limited sample size within each stratum, these stratified analyses should be interpreted as exploratory.

### Comparison of Ferritin, PCT, CRP, and Serum Cr Levels between ST and Brucella Meningitis

**Fig 3:**
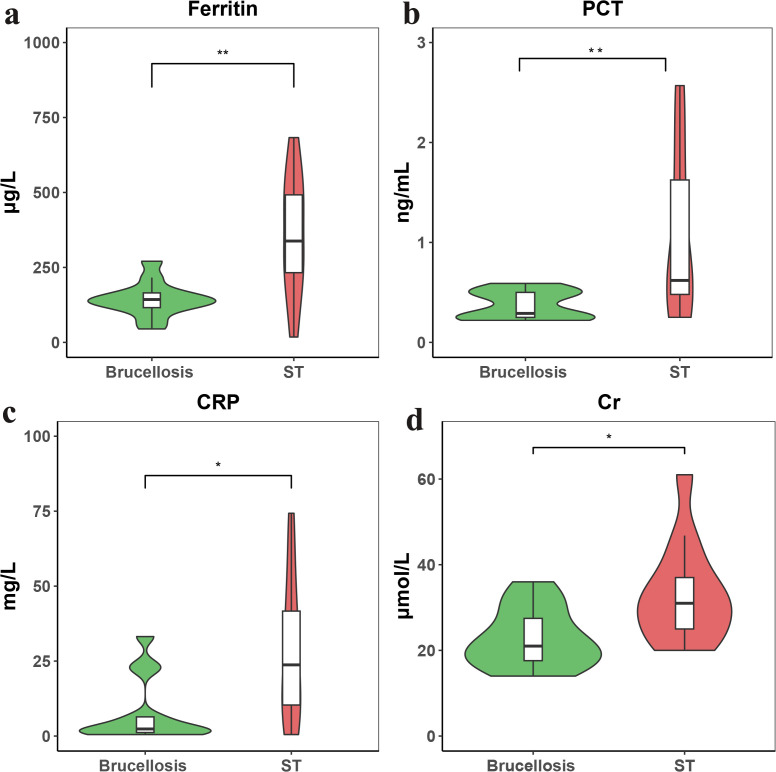
Comparison of clinical biomarkers for Brucellosis and Scrub Typhus meningitis. **(a)** Ferritin (µg/L), **(b)** Procalcitonin (PCT, ng/mL), **(c)** C-Reactive Protein (CRP, mg/L), **(d)** Serum Creatinine (Cr, µmol/L).

The ROC curve analysis highlighted the diagnostic significance of ferritin, PCT, CRP, and serum creatinine in differentiating typhus meningitis from Brucella meningitis. To further illustrate these findings, bar graphs were generated to compare the levels of these four biomarkers between patients with ST meningitis and those with Brucella meningitis **(see Fig 3).**

The results indicated that the levels of ferritin, PCT, CRP, and Cr were all significantly higher in the ST group compared with the Brucella group. Notably, ferritin and PCT levels were markedly elevated in the ST group, with differences reaching high statistical significance.

## Discussion

This study retrospectively analyzed pediatric cases of brucellar meningitis admitted to Kunming Children’s Hospital over the past decade, using the propensity score matching method to select an equal number of ST meningitis cases for comparison. By systematically evaluating the clinical features, laboratory parameters, and CSF findings, the study demonstrated that although both diseases can cause meningitis in children and present with prolonged fever, they differ significantly in hematological and certain biochemical indicators. Children with ST meningitis exhibited markedly higher levels of WBC, Cr, CRP, PCT, and ferritin compared with those with brucellar meningitis. These findings suggest that these parameters may provide preliminary indications for early differentiation; however, their diagnostic performance should be interpreted with caution due to the small sample size after matching. ROC and DCA further suggest a potential signal that ferritin and PCT could be explored as candidate markers in future studies.

The CSF analyses revealed that, apart from elevated cell counts, glucose, chloride, and albumin levels in both groups remained within normal ranges, which was consistent with previous study [17]. This suggests that the underlying pathophysiology in both diseases is more likely related to mild meningeal inflammation rather than the exudative changes typically seen in bacterial or tuberculous meningitis [18]. Since both ST and brucellosis are caused by intracellular pathogens, the central nervous system manifestations may result predominantly from immune-mediated inflammatory responses and cytokine release, rather than direct tissue destruction [13, 19]. Accordingly, the relatively mild CSF biochemical profile may help distinguish these infections from tuberculous meningitis (which usually presents with hypoglycorrhachia, hypochloremia, and elevated protein) and from purulent meningitis (characterized by marked pleocytosis, high protein, and low glucose levels).Clinically, both groups of patients presented with fever, but typical features were often absent. Eschar formation, a hallmark of ST, was not consistently observed, while some patients with brucellosis lacked joint pain or a history of animal contact [20]. These overlapping clinical patterns make early differentiation particularly challenging. Meningeal symptoms such as headache, vomiting, and lethargy showed no significant differences between the groups. However, the duration of fever was notably longer in brucellosis. One possible explanation is the intracellular persistence of Brucella, which allows the pathogen to survive within the mononuclear phagocyte system and trigger chronic immune activation. This may lead to persistent or relapsing fever [21, 22]. In contrast, ST typically has a shorter disease course but can rapidly progress to multiorgan dysfunction or shock if untreated, necessitating intensive care [23]. Therefore, differences in fever duration may serve as a useful clinical clue for differentiation.

The study found significant intergroup differences in ferritin, PCT, CRP, and Cr. However, these markers should be interpreted cautiously because they are non-specific across a broad range of infectious and inflammatory conditions. Elevated ferritin reflects heightened systemic inflammation and macrophage activation, as well as disrupted iron metabolism [24, 25]. ST, as an acute Rickettsial infection, tends to induce a stronger systemic inflammatory response and cytokine release, often leading to markedly increased ferritin levels. In ST, Orientia tsutsugamushi triggers robust activation of macrophages and endothelial cells.This process is accompanied by increased pro-inflammatory cytokines, such as IFN-γ, TNF-α, and IL-6. This pattern of immune activation contributes to the pronounced ferritin elevation observed in ST meningitis. Previous studies [26, 27] have also reported ST-associated hemophagocytic lymphohistiocytosis (HLH). Together, these findings raise the possibility that ST may induce an HLH-like hyperinflammatory response, characterized by macrophage overactivation and excessive cytokine release. This mechanism may also help explain the concurrent rise in ferritin and PCT in our cohort. In contrast, *Brucella* infection typically induces a slower and less intense inflammatory response, resulting in modest ferritin elevation. Nevertheless, ferritin elevation is not unique to ST and can also be seen in other infections including viral meningitis where ferritin has been reported as a poor discriminator, limiting its specificity. From a pragmatic perspective, ferritin is relatively widely available in routine clinical laboratories, whereas PCT testing may be less accessible and more costly in some resource-limited settings; therefore, local feasibility and affordability should be considered when applying these biomarkers.

PCT and CRP are acute-phase reactants, and their higher levels in ST likely reflect more pronounced production of inflammatory mediators such as interleukin-6 and tumor necrosis factor-α [28, 29]. Compared with brucellosis, ST generally results in a more abrupt and intense cytokine cascade, which may underlie the more significant increases in PCT and CRP. Similar trends have also been observed in other rickettsial infections, further supporting this interpretation [30]. Yet, similar to ferritin, both PCT and CRP can be elevated in various bacterial and viral infections, and thus their discriminatory ability between ST and brucellar meningitis should be regarded as exploratory rather than definitive.

The elevation of serum Cr may indicate renal involvement due to tubular injury or impaired perfusion secondary to ST infection [31]. However, in the absence of urinalysis or estimated glomerular filtration rate (eGFR) data, this interpretation remains speculative. Collectively, these findings suggest that ferritin and PCT are not only markers of inflammation severity but may also serve as biological indicators distinguishing between the two types of infection. This study provides preliminary observational evidence regarding the potential diagnostic value of these markers in differentiating ST from brucellar meningitis in children. However, because we required a CSF cell count above the diagnostic threshold for meningitis, early-stage or low-cell-count meningitis cases were excluded. This means that our findings primarily reflect children with established lymphocytic meningitis and may not be generalizable to early or atypical clinical presentations.

ST meningitis typically requires doxycycline as first-line therapy, whereas brucellar meningitis requires prolonged combination therapy such as rifampin plus doxycycline, with or without ceftriaxone to achieve adequate central nervous system penetration and prevent relapse. In this context, diagnostic biomarkers such as markedly elevated ferritin or PCT may help clinicians favor a diagnosis of ST rather than brucellosis, thereby supporting earlier initiation of doxycycline and avoiding delays in effective treatment. Although both diseases can affect the central nervous system, all patients in this study showed clinical improvement after timely diagnosis and standardized treatment, with no cases of recurrence or death. This highlights that early recognition and appropriate antimicrobial therapy can lead to favorable outcomes in pediatric ST and brucellar meningitis. Prompt diagnosis and intervention are essential to shorten the disease course and reduce neurological sequelae, thereby improving prognosis.

Several limitations of this study should be acknowledged. First, it was a single-center retrospective analysis with a relatively small sample size. Given that only 13 pediatric brucellar meningitis cases were identified in 10 years, the study is substantially underpowered, and no a priori power calculation was performed, which further limits the interpretability of the statistical findings. the small sample size limits the stability and reliability of the statistical estimates. Second, all cases were derived from hospitalized pediatric patients, lacking representation of mild community cases, which may introduce selection bias. Furthermore, the ROC performed in this study may be prone to overfitting due to the limited sample size, and therefore the diagnostic performance observed should be interpreted as exploratory rather than confirmatory. Additionally, because diagnostic criteria for brucellar meningitis can vary in clinical practice, the possibility of diagnostic misclassification cannot be excluded. Multiple univariate comparisons were conducted without adjustment, which increases the risk of type I error. In addition, dynamic changes in biomarkers and long-term follow-up data were not analyzed. These limitations underscore that the findings should be viewed as preliminary observations that require external validation. Future studies should adopt more specific methodological approaches, including multicenter prospective designs with adequately powered sample sizes (e.g., ≥ 100 patients per group for reliable ROC analysis), standardized timing of biomarker collection, and external validation of proposed cutoff values for ferritin, PCT, and other indicators to better determine their true diagnostic utility in differentiating ST meningitis from brucellar meningitis.

## Conclusions

Although scrub typhus meningitis and brucellar meningitis share considerable clinical overlap in children, serum ferritin and PCT levels may represent potential diagnostic signals for early differential diagnosis, warranting validation in larger prospective cohorts. High Ferritin levels or PCT levels may provide preliminary clues toward ST meningitis. Early recognition and targeted antimicrobial therapy are associated with favorable prognostic outcomes.

## Supporting information

S1 Table Summary of Diagnostic Criteria for Scrub Typhus (ST) and Brucellosis Used in This Study. (DOCX)

S2 Table The original data used in this study for analysis. (XLSX)
