# Supplementary material for: Differentiating scrub typhus meningitis from brucellar meningitis in children: A single-center retrospective study
Source: PLoS Negl Trop Dis. 2026 Mar 13;20(3):e0014107. doi: 10.1371/journal.pntd.0014107 (PMC12987469; doi:10.1371/journal.pntd.0014107)
Supplement: S1 Table — (DOCX) [file pntd.0014107.s001.docx]

**Table 1.** Summary of Diagnostic Criteria for Scrub Typhus (ST) and Brucellosis Used in This Study.

| **Item** | **Scrub Typhus (ST)** | **Brucellosis** |
| --- | --- | --- |
| **Diagnostic Guidelines Used** | Pediatric Infectious Diseases Diagnostic Criteria for ST (5th edition) | Diagnosis and Treatment Scheme for Brucellosis (2023 Edition) |
| **Inclusion Criteria – Clinical** | ● Acute fever with history of outdoor exposure within 3 weeks in endemic area  ● Presence of typical signs: eschar/ulcer, rash, lymphadenopathy | ● Compatible clinical manifestations suggestive of brucellosis |
| **Inclusion Criteria – Laboratory / Etiological Tests** | **At least one of the following:**  ● Specific IgM positive  ● Fourfold rise in antibody titers (Weil-Felix)  ● **PCR positive (required for inclusion in this study)** | **Etiological Diagnosis:**  ● Isolation of Brucella spp. from blood, bone marrow, CSF, or other specimens  **Initial Screening Tests:**  ● Rose Bengal Test (RBT) positive  ● GICA positive  ● ELISA positive  **Confirmatory Serological Tests:**  ● SAT titer ≥1:100 (or ≥1:50 if symptoms >1 year)  ● CFT titer ≥1:10  ● Coombs test titer ≥1:160400 |
| **Case Definition Used in This Study** | Only PCR-positive cases were included; no patient was diagnosed based solely on IgM. | Only cases fulfilling “Confirmed Diagnosis” were included; screening-positive suspected cases were excluded. |
| **Exclusion Criteria** | ● Congenital heart disease, hematologic malignancies, immunodeficiency  ● Incomplete clinical data  ● Normal CSF cell count | ● Co-infection with other acute infections  ● Incomplete clinical data  ● Congenital heart disease, hematologic malignancies, immunodeficiency  ● Normal CSF cell count |
